# Environmental DNA reveals temporal variation in mesophotic reefs of the Humboldt upwelling ecosystems of central Chile: Toward a baseline for biodiversity monitoring of unexplored marine habitats

**DOI:** 10.1002/ece3.10999

**Published:** 2024-02-21

**Authors:** Pablo Saenz‐Agudelo, Paula Ramirez, Ricardo Beldade, Ana N. Campoy, Vladimir Garmendia, Francesca V. Search, Miriam Fernández, Evie A. Wieters, Sergio A. Navarrete, Mauricio F. Landaeta, Alejandro Pérez‐Matus

**Affiliations:** ^1^ Instituto de Ciencias Ambientales y Evolutivas, Universidad Austral de Chile Valdivia Chile; ^2^ Millennium Nucleus for Ecology and Conservation of Temperate Marine Ecosystems, NUTME Las Cruces Chile; ^3^ Estación Costera de Investigaciones Marinas Pontificia Universidad Católica Las Cruces Chile; ^4^ Center of Marine Sciences (CCMAR‐CIMAR) University of the Algarve Faro Portugal; ^5^ Center for Applied Ecology and Sustainability (CAPES) and Coastal Socio‐Ecological Millennium Institute (SECOS) Pontificia Universidad Católica de Chile Santiago Chile; ^6^ Center for Oceanographic Research COASTAL‐COASTAL Universidad de Concepción Concepción Chile; ^7^ Laboratorio de Ictiología e Interacciones Biofísicas (LABITI) Instituto de Biología, Facultad de Ciencias, Universidad de Valparaíso Valparaíso Chile

**Keywords:** biodiversity survey, eDNA, eukaryotes, fishes, metabarcoding

## Abstract

Temperate mesophotic reef ecosystems (TMREs) are among the least known marine habitats. Information on their diversity and ecology is geographically and temporally scarce, especially in highly productive large upwelling ecosystems. Lack of information remains an obstacle to understanding the importance of TMREs as habitats, biodiversity reservoirs and their connections with better‐studied shallow reefs. Here, we use environmental DNA (eDNA) from water samples to characterize the community composition of TMREs on the central Chilean coast, generating the first baseline for monitoring the biodiversity of these habitats. We analyzed samples from two depths (30 and 60 m) over four seasons (spring, summer, autumn, and winter) and at two locations approximately 16 km apart. We used a panel of three metabarcodes, two that target all eukaryotes (18S rRNA and mitochondrial COI) and one specifically targeting fishes (16S rRNA). All panels combined encompassed eDNA assigned to 42 phyla, 90 classes, 237 orders, and 402 families. The highest family richness was found for the phyla Arthropoda, Bacillariophyta, and Chordata. Overall, family richness was similar between depths but decreased during summer, a pattern consistent at both locations. Our results indicate that the structure (composition) of the mesophotic communities varied predominantly with seasons. We analyzed further the better‐resolved fish assemblage and compared eDNA with other visual methods at the same locations and depths. We recovered eDNA from 19 genera of fish, six of these have also been observed on towed underwater videos, while 13 were unique to eDNA. We discuss the potential drivers of seasonal differences in community composition and richness. Our results suggest that eDNA can provide valuable insights for monitoring TMRE communities but highlight the necessity of completing reference DNA databases available for this region.

## Graphical abstract

Here, environmental DNA (eDNA) was used to characterize temperate mesophotic reef ecosystems (TMREs) along the central Chilean coast, shedding light on their biodiversity. Through eDNA analysis at different depths and seasons, we identified 42 phyla and 237 orders, with Arthropoda, Bacillariophyta, and Chordata being the most diverse. Notably, TMRE community composition varied seasonally, particularly in summer, emphasizing the potential of eDNA for monitoring these ecosystems and the need to enhance reference DNA databases for the region.

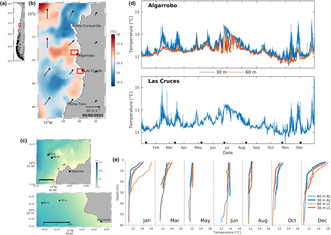

## 1 INTRODUCTION

Marine ecosystems provide numerous services to human populations around the world (Liquete et al., 2013). Surveying and understanding how biodiversity is distributed in space and time is essential to implement actions to manage, preserve, monitor, and restore marine ecosystems. Among different marine ecosystems, mesophotic reefs have gained research interest over the last decades due to technological advances that have led to safer, deeper explorations of the coastal oceans (Rocha et al., 2018). Mesophotic reefs are hard‐bottom marine ecosystems found in the dimly lit, middle zone of the coastal oceans, typically between depths of 30 and 150 m (Eyal & Pinheiro, 2020; Rocha et al., 2018), where light becomes limiting and down to about 1% of the surface irradiance (Cerrano et al., 2019). Other authors argue that these ecosystems are composed of differentiated communities that must be defined according to regionally variable depth boundaries and local environmental conditions (Bell et al., 2022; Pérez‐Castro et al., 2022). Current knowledge about the biodiversity associated with these reefs remains very limited compared to shallow reefs (Soares et al., 2018; Turner et al., 2019). Still, over the last decade, substantial research on these ecosystems has begun to show that they might act as ecological corridors and refugia and harbor novel species or genes (Kahng et al., 2017; Soares et al., 2020).

Temperate mesophotic reef ecosystems (TMREs) have received less attention than their tropical counterparts. However, research is increasing in different parts of the world, and recent findings reveal that they constitute essential habitats for diverse marine organisms (Bell et al., 2022; Cerrano et al., 2019). In this regard, these ecosystems can be critical in preserving marine biodiversity and sustaining demersal/benthic fisheries (Landaeta et al., 2023). Moreover, TMREs have also been suggested as potential refugia for fish species and other organisms in the face of climate change and other environmental stressors that occur in coastal surface waters (Assis et al., 2016; Cerrano et al., 2019). However, this idea has been questioned for tropical mesophotic coral reefs (Medeiros et al., 2021; Rocha et al., 2018; Smith et al., 2016; Soares et al., 2018). Whether or not TMREs are effective refuges and maybe more, instead of less, exposed to disturbances and climate‐driven fluctuations remain to be evaluated. This knowledge gap emphasizes the urgent need to devise effective monitoring programs to document their potentially rapid transformation. However, due to the inherent limitations of the unknown aspects of TMREs, delving deeper into their definition, distribution, and the biodiversity they support are crucial in developing effective management strategies to safeguard and conserve these vital marine ecosystems (Turner et al., 2019).

TMREs in the South‐Eastern Pacific are notably understudied compared to other regions. According to a recent review (Bell et al., 2022), most studies on TMREs have been conducted in the Mediterranean Sea, Temperate Australasia, the North Pacific, and the North Atlantic oceans. Only two studies document the broad structure of benthic communities near the upper limit of mid‐depths (30 m) (Betti et al., 2017) or across mid‐depths encompassing mesophotic reefs (down to 100 m) in the South‐Eastern Pacific (Campoy et al., 2023). The Chilean coast is one of the most productive coastal ecosystems on the planet (Carr & Kearns, 2003; Thiel et al., 2007), fueled by wind‐driven upwelling that brings cold, salty, nutrient‐rich, and oxygen‐poor water inshore. Along the central coast, upwelling‐favorable winds are distinguished by seasonal fluctuations such that upwelling events are concentrated in spring‐summer and locally modified by coastal topography, driving important spatiotemporal variation in surface seawater temperature and surface water plankton (Weidberg et al., 2020; Wieters et al., 2003), as well as in the dynamics and structure of intertidal and shallow subtidal populations and communities (Lurgi et al., 2020; Navarrete et al., 2005; Pérez‐Matus et al., 2017; Wieters et al., 2009). However, scarce information exists below 30 m depth regarding the seasonality of TMRE community composition and the spatial scales of variation among sites or across the depth gradient. The only quantitative characterization of mesophotic reefs here is a recent study describing macroscopic invertebrate and fish species using towed underwater videos (Campoy et al., 2023). Bycatch fisheries data associated with crustacean trawling fisheries, which are conducted mostly over the soft bottom of the continental shelf, have also provided a coarse characterization of mesophotic and deeper water assemblages (Landaeta et al., 2023; Montero et al., 2020).

Environmental DNA (eDNA) metabarcoding is rising as a powerful tool for assessing and monitoring biodiversity in marine ecosystems. eDNA metabarcoding is a non‐invasive, less time‐consuming, and cost‐effective technique than other traditional sampling strategies (Deiner et al., 2017). Although technological advances such as remote‐operated vehicles (ROVs) or rebreather scuba diving have become more accessible, their repeated use over extensive geographic areas in deep habitats remains challenging and costly. Thus, despite current challenges and limitations with regard to process and scale uncertainties (e.g. Hansen et al., 2018), eDNA can be an effective integrational tool for implementing biodiversity monitoring programs. For instance, eDNA has been used to characterize subarctic deep‐water fishes (Thomsen et al., 2016) and deep‐sea benthic biodiversity (Brandt et al., 2021; Liu & Zhang, 2021). Several studies have also used this tool to assess biodiversity in shallow reefs (DiBattista et al., 2020; Dugal et al., 2022; Ip et al., 2022; Marwayana et al., 2022; Oka et al., 2021; Polanco Fernández et al., 2021). To our knowledge, only a couple of studies have successfully used eDNA to document mesophotic reef diversity. One evaluated fish diversity in one tropical and one temperate mesophotic reef highlights the large diversity of fish species identified at the two study sites and especially at mid‐depths (Muff et al., 2023). The other one used three metabarcodes to evaluate fish, eukaryote, and metazoan diversity in a tropical mesophotic reef (Hoban et al., 2023). The general extent of these patterns in productive upwelling temperate reefs and in other groups of marine organisms has not yet been assessed.

Here, we report the first eDNA eukaryote biodiversity survey of TMREs from the coast of central Chile. We examined whether there are seasonal differences in community composition, whether there are differences between depths (30, 60 m), and whether these patterns are persistent between sites separated by 16 km and subjected to variable seasonal environmental conditions. We use two “Universal” metabarcoding essays (18S rRNA and COI) and a fish‐specific assay targeting the 16S ribosomal RNA (16S rRNA) to characterize eukaryote biodiversity. This study represents the first biodiversity survey of mesophotic reefs in upwelling regions using eDNA metabarcoding. These data will contribute to the growing body of literature assessing the utility of this methodology in the development of biodiversity monitoring programs.

## 2 METHODS

### 2.1 Study area

**FIGURE 1:**
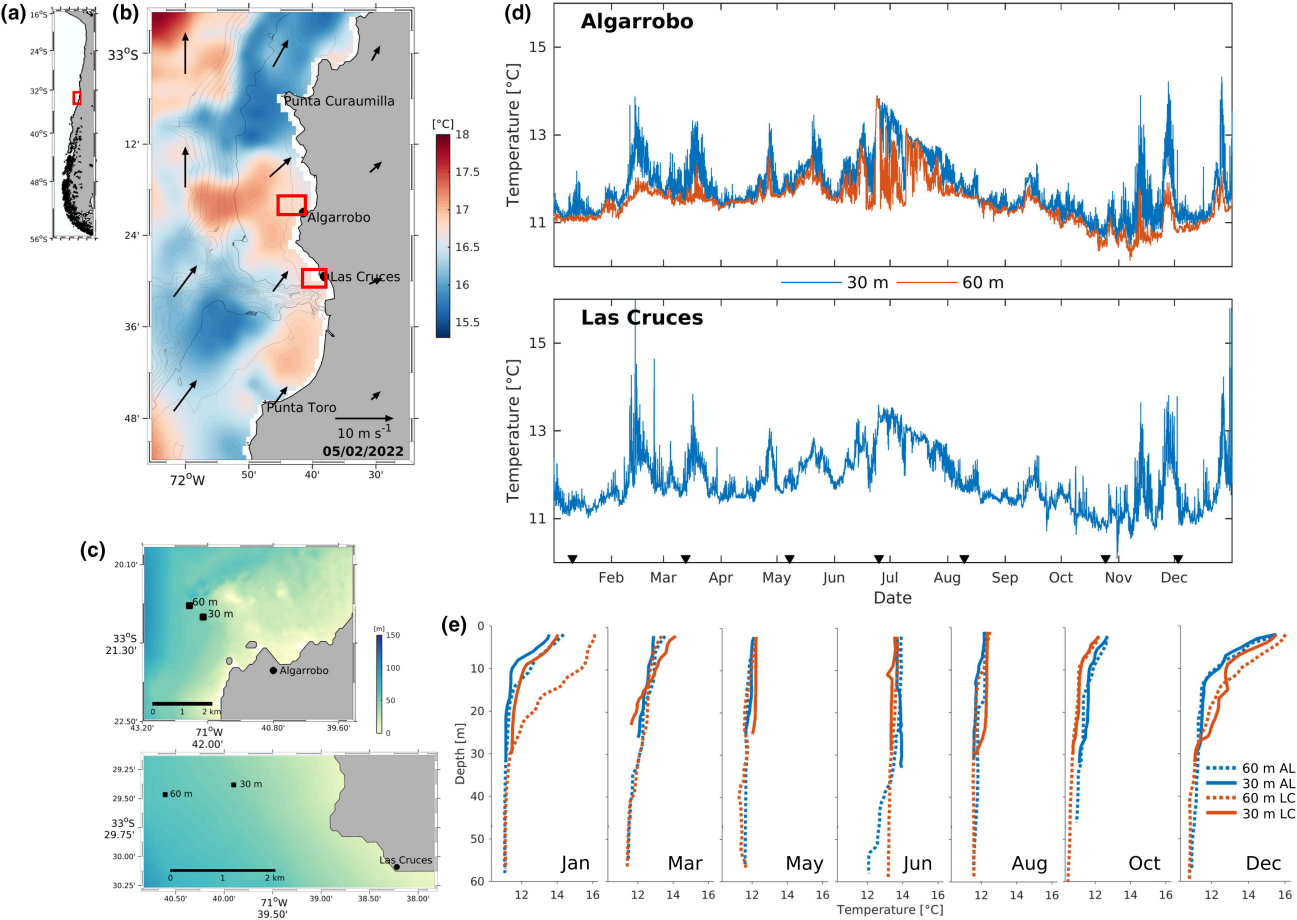
(a) Map of the South‐Eastern Pacific coast of South America. (b) Coast of central Chile, indicating the two study locations, with MUR sea surface temperature (SST) satellite data during February 5, 2022, showing upwelling intensification produced by equatorial winds (arrows, satellite‐derived winds ERA5), here visible by colder surface waters at Punta Curaumilla (PC) and at Punta Toro (PT). (c) Location of 30 and 60 m sensors and eDNA surveys. (d) Bottom temperatures at study sites during 2022. eDNA surveys are marked with black triangles at the bottom of panel d and temperatures between sites were highly correlated (Pearson *r* > 0.9). (e) Temperature profiles for each eDNA survey. Note a well‐defined seasonal thermocline during the summer months (Jan, Dec), which was weaker in fall (March) and spring (October), and nonexistent in winter. Additionally, temperatures below 30 m remained between 10.5 and 14°C throughout the year, with occasional brief (>1 h) peaks that reached above 14°C at Las Cruces. AL: Algarrobo, LC: Las Cruces.

The survey sites were located off Algarrobo (33.365° S, 71.671° W) and Las Cruces (33.498° S, 71.623° W) in the Valparaíso region of the central Chile. These sites were chosen because of their easy accessibility and reduced logistic constraints for preparing and executing sampling expeditions. Las Cruces is located at the end of Cartagena Bay, and has been described in previous studies as an upwelling shadow (Bonicelli et al., 2014; Largier, 2020). Using satellite‐derived sea surface temperatures (SST) as a proxy for upwelling (Figure 1b), both sites were observed to have relatively similar upwelling exposure and environmental characteristics (Figure 1b). Observations with SCUBA diving and towed underwater videos were used to confirm the presence of rocky substrate and choose the sites for water sampling (Figure 1c). At each site, bottom‐mounted oxygen‐temperature loggers (PME MiniDOT) were deployed at 30 m and 60 m depths with 10 and 20‐min sampling intervals, respectively. Unfortunately, the 60 m Las Cruces sensor was not recovered. Additionally, during each eDNA survey, physical water parameters (salinity and temperature) were sampled using an SBE 19 CTD, from the surface to approximately 1 m above the bottom. A summary of these environmental data is presented in Figure 1d,e to provide the context for the eDNA surveys and allow comparison with future studies.

### 2.2 eDNA sampling

Water sampling for eDNA analyses was conducted at each site with samples taken at 30 m and 60 m depths using 2.5 L Niskin bottles deployed from a small research vessel. Water samples were consistently taken at 1 m above the rocky substrate. Water samples at each site and depth were collected twice seasonally between January and December 2022 (Table 1). During the winter season, only one sampling event was conducted due to adverse weather conditions. In each sampling event, three 1 L sample replicates were collected per depth, and two negative controls (one control consisting of filtering 0.5 L of pure water, and the other control consisting of filtering 0.5 L of the bleach solution used to disinfect filtering material). Samples were filtered on board using a modified manual and peristaltic vacuum pump with 0.45 μm mixed cellulose ester membrane (Merck Millipore Corp., Billerica, MA, USA). The filters were stored in 1.5 mL plastic tubes that contained 1 mL of lysis buffer (Thermo Fisher Scientific, Waltham, MA, USA) and kept at 4°C until extraction. The Niskin bottle and all filtration equipment used for eDNA sampling were rinsed using 2.5% bleach between samples.

**TABLE 1:** Sampling details of eDNA samples for this study.

Code	Replicates	Volume filtered (L)	Depth (m)	Site	Season	Longitude	Latitude	Date
ALG30_V1	3	1	30	Algarrobo	Summer	−71.701	−33.349	12/1/2022
ALG60_V1	3	1	60	Algarrobo	Summer	−71.706	−33.345	12/1/2022
LC30_V1	3	1	30	Las Cruces	Summer	−71.665	−33.490	11/1/2022
LC60_V1	3	1	60	Las Cruces	Summer	−71.678	−33.491	11/1/2022
ALG30_V2	3	1	30	Algarrobo	Summer	−71.701	−33.349	14/3/2022
ALG60_V2	3	1	60	Algarrobo	Summer	−71.706	−33.345	14/3/2022
LC30_V2	3	1	30	Las Cruces	Summer	−71.665	−33.490	13/3/2022
LC60_V2	3	1	60	Las Cruces	Summer	−71.678	−33.491	13/3/2022
ALG30_OT1	3	1	30	Algarrobo	Autumn	−71.701	−33.349	9/5/2022
ALG60_OT1	3	1	60	Algarrobo	Autumn	−71.706	−33.345	9/5/2022
LC30_OT1	3	1	30	Las Cruces	Autumn	−71.665	−33.490	8/5/2022
LC60_OT1	3	1	60	Las Cruces	Autumn	−71.678	−33.491	8/5/2022
ALG30_OT2	3	1	30	Algarrobo	Autumn	−71.701	−33.349	26/6/2022
ALG60_OT2	3	1	60	Algarrobo	Autumn	−71.706	−33.345	26/6/2022
LC30_OT2	3	1	30	Las Cruces	Autumn	−71.665	−33.490	25/6/2022
LC60_OT2	3	1	60	Las Cruces	Autumn	−71.678	−33.491	25/6/2022
ALG30_INV	3	1	30	Algarrobo	Winter	−71.701	−33.349	11/8/2022
ALG60_INV	3	1	60	Algarrobo	Winter	−71.706	−33.345	11/8/2022
LC30_INV	3	1	30	Las Cruces	Winter	−71.665	−33.490	10/8/2022
LC60_INV	3	1	60	Las Cruces	Winter	−71.678	−33.491	10/8/2022
ALG30_PR1	3	1	30	Algarrobo	Spring	−71.701	−33.349	26/10/2022
ALG60_PR1	3	1	60	Algarrobo	Spring	−71.706	−33.345	26/10/2022
LC30_PR1	3	1	30	Las Cruces	Spring	−71.665	−33.490	25/10/2022
LC60_PR1	3	1	60	Las Cruces	Spring	−71.678	−33.491	25/10/2022
ALG30_PR2	3	1	30	Algarrobo	Spring	−71.701	−33.349	3/12/2022
ALG60_PR2	3	1	60	Algarrobo	Spring	−71.706	−33.345	3/12/2022
LC30_PR2	3	1	30	Las Cruces	Spring	−71.665	−33.490	3/12/2022
LC60_PR2	3	1	60	Las Cruces	Spring	−71.678	−33.491	3/12/2022
Water_cntrl	13	0.5	Negative controls			–	–	–
Bleach_cntrl	13	0.5	Negative controls			–	–	–

### 2.3 Laboratory procedures

#### 2.3.1 eDNA extractions

Before DNA extraction, each 1.5 mL tube containing the filters and lysis buffer was shaken using a Mini Beadbeater (BioSpec Products Inc., Bartlesville, USA) for 1 min at 2.5 × 1.000 stroke/min. Then, within a laminar flow chamber, 500 μL of the lysis buffer was transferred into a 1.5 mL microcentrifuge tube for DNA extraction. DNA was then extracted and purified using the GeneJET Genomic DNA Purification Kit (Thermo Fisher Scientific, Waltham, MA, USA), following the manufacturer's instructions with the following modifications: (1) 50 μL of the Proteinase K solution was added to the 500 mL containing eDNA and lysis buffer, and then vortexed; (2) samples were then incubated at 56°C for 3 h; (3) 500 μL of 50% ethanol was added. DNA samples were eluted in 60 μL of elution buffer. DNA extractions were processed in batches of 11 samples, and for each batch, a negative extraction control (nine in total) was included (blank DNA extraction). Purified extracted DNA samples were stored at −20°C until PCR amplification.

#### 2.3.2 PCR amplification

eDNA amplification was done using a two‐step PCR method, to amplify eDNA and add unique combinations of dual barcodes to each sample. DNA samples, including field and laboratory negative controls, were first amplified with three primer sets targeting specific fragments of mitochondrial (COI) and rRNA genes (18S and 16S). These primers included an Illumina primer sequence, a 12‐base pair (bp) barcode with a 0–4 bp spacer, and a specific targeted primer sequence (Table 2). In the second PCR step, pools of samples were amplified to incorporate specific Illumina labeling indexes for each library. Besides the field negative controls (water control *N* = 13, and bleach control *N* = 13), a total of nine negative extraction controls and four negative PCR controls per metabarcoding were also included.

**TABLE 2:** Details regarding the primer pairs employed for metabarcoding in this study.

Primer name	Sequence	Target gene	Average PCR length	Reference
16SF/D 16S2R‐deg	GACCCTATGGAGCTTTAGAC CGCTGTTATCCCTADRGTAACT	16S rRNA gene	202	Berry et al. (2017) Deagle et al. (2007)
mlCOIInt jgHCO	GGWACWGGWTGAACWGTWTAYCCYCC TAIACYTCIGGRTGICCRAARAAYCA	COI gene	313	Leray et al. (2013)
SSU_FO4 SSU_R22	GCTTGTCTCAAAGATTAAGCC GCCTGCTGCCTTCCTTGGA	18S rRNA gene	450	Fonseca et al. (2010)

For each sample, the first PCR reaction was performed in duplicate (technical PCR replicate) for each primer. The reaction mixes consisting of 6 μL of AmpliTaq GoldTM Fast PCR Master Mix (Illumina, San Diego, CA, USA), 1 μL forward primer (2.5 μM), 1 μL reverse primer (2.5 μM), 0.5 μL BSA (10 mg/mL), 2.5 μL DNA template and 1.5 μL H_2_O to reach a final volume of 12.5 μL. The thermal cycling conditions followed with annealing temperatures depending on primer pairs: 54°C for the 16S, 52°C for 18S and 50°C for COI. After an initial denaturation step of 7 min at 95°C, each of the 40 cycles consisted of 30s at 95°C, 30s at the primer‐specific annealing temperatures as described above, and 30s at 72°C, with a final extension of 10 min at 72°C.

The same barcode pair was used for all PCR reactions corresponding to replicates from the same sample (three replicates per sample). The amplicon sizes of each reaction were visualized on a 1.5% agarose gel to confirm amplification quality. For the field samples, the amplicons obtained from the first PCR were then pooled per sample (for each sample, six PCR products were pooled, corresponding to three biological replicates and two technical replicates per sample). Negative controls were not pooled together. Thus, for the bioinformatic analyses, the complete dataset included 28 field samples and 26 negative control samples. Sampling details can be found in Table 1.

Preparation of all PCR reactions was performed in a laminar flux chamber following sterilization. Each pooled PCR product was then cleaned using the Agencourt AMPure XP bead system (Beckman Coulter, USA) to remove primer dimers by size selection. Following purification, the clean pooled PCR products were assessed for DNA concentration using the QUBIT DS DNA HS assay (Life Technologies, Waltham, MA, USA). Subsequently, the pools of PCR products obtained with the same primer set were combined and all PCR products were standardized. A portion of this pool was then utilized in a second PCR to incorporate Illumina‐specific sequencing indexes. The second PCR was carried out in a 50 μL volume, comprising 5 μL of DNA template, 5 μL of Nextera XT Index Primer 1, 5 μL of Nextera XT Index Primer 2 (Illumina, San Diego, CA, USA), 25 μL of 2× KAPA HiFi HotStart Ready Mix, and 10 μL of H_2_O. The thermocycling profile consisted of an initial denaturation step at 95°C for 3 min, followed by eight cycles of denaturation at 95°C for 30 s, annealing at 55°C for 30 s, and extension at 72°C for 30 s, with a final extension at 72°C for 5 min. Each library was subjected to purification using AMPure beads, quantification using a fluorometric procedure (Qubit 3.0 Fluorometer; Life Technologies), and amplification verification using capillary electrophoresis in a Fragment Analyzer System (Agilent Technologies, USA). Subsequently, all libraries (three per sequencing run) were combined in an equal molar ratio. These libraries were then subjected to sequencing on an Illumina MiSeq platform (Illumina, San Diego, CA) at the AUSTRAL‐omics bioscience core facility, utilizing a concentration of 12 pM, 250‐bp paired‐end sequencing, and including a 20% Phi‐X spike‐in control.

### 2.4 Bioinformatics procedures

We performed demultiplexing of Illumina sequences using Geneious Prime 2022.0.2. Then, the Anacapa pipeline (Curd et al., 2019) was employed for data processing, including reference database creation, quality control, amplicon sequence variant parsing, and taxonomic assignment. Default settings were used for all pipeline steps. Custom CRUX reference libraries were created for each primer set following Curd et al. (2019) recommendations. We then ran the Anacapa pipeline “Anacapa_QC_dada2.sh” on the demultiplexed fastq files to quality filter sequence data and to generate Amplicon Sequence Variants (ASV) (Callahan, Proctor, et al., 2016). Briefly, this pipeline takes each demultiplexed fastq file, passes it through cutadapt (Martin, 2011) to quality trim sequences, and removes adapters. Then Fast‐toolkit was used to remove low‐quality reads (*Q* < 30 were removed) and sort reads by primer sequence (Gordon & Hannon, 2010). Reads are then sorted and input into DADA2, merged (if possible), denoised, tested for chimeric sequences, and grouped into ASVs (Callahan, McMurdie, et al., 2016). Any sequence had to occur at least four times to be retained as an ASV. We then ran the “Anacapa_classifier.sh” to assign taxonomy to each of the ASVs. For this, the algorithm uses Bowtie2 (Langmead & Salzberg, 2012) to align ASVs to the reference catalog and record the best 100 hits. These results were then input into Bootstrap Confidence‐Limited Assignments (BCLA) (Gao et al., 2017), where bootstrap confidence scores were estimated for taxonomic assignments. The output of this procedure consisted of summary tables of ASVs and taxonomy count data for each sample and primer set. For further analyses, we used taxonomic assignments with a BCLA confidence score 0.8 or higher. This threshold was chosen as a conservative measure to balance assignment accuracy and misclassification rates (Curd et al., 2019; Gold et al., 2021).

#### 2.4.1 Data cleaning and statistical analyses

We imported the resulting taxonomic tables per metabarcode into R v 3.6.3 (R Core Team, 2018) and transformed them into *phyloseq* objects (McMurdie & Holmes, 2013). Then, we used the *decontam* R package (Davis et al., 2018) to remove contaminant ASVs that appeared in negative controls (*N* = 39). We applied the prevalence method with a threshold of 0.5. We also manually removed sequences assigned to non‐marine taxa from the dataset (e.g. terrestrial mammals). After contaminant removal, *phyloseq* objects obtained from the three different metabarcodes (18s, 16s and COI) were merged for further statistical analyses. Rarefaction curves were generated to ensure adequate sequencing depth. Data were rarefied to the minimum number of reads per sample (112,203) using the “rarefy_even_depth” function in the *Phyloseq* package (McMurdie & Holmes, 2013).

For alpha diversity proxies, observed richness was estimated and plotted according to depth, site, and season using the “estimate_richness” and “ggplot” functions from the *Phyloseq* (McMurdie & Holmes, 2013) and *ggplot2* (Wickham, 2011) packages, respectively. The Kruskal‐Wallis test from the *stats* package (R Core Team, 2018) was used to compare the medians of observed richness based on these categories. To test for differences in the dissimilarity of community composition (with season, depth, and site as explanatory variables) a Multifactorial Permutational Analysis of Variance (PERMANOVA) was performed using the “adonis2” function in the vegan package (Oksanen et al., 2019). The PERMANOVA was conducted on community matrices with Bray‐Curtis dissimilarities created using the “distance” function in the *phyloseq* package (McMurdie & Holmes, 2013). The presence and absence data at the family level were used to generate the dissimilarity matrices (similar trends were obtained when ASVs were used to estimate dissimilarity matrices, but only family‐level results are presented). To illustrate differences in TMRE community composition per depth, site, and seasons, the Bray–Curtis dissimilarity matrices were ordinated using a non‐metric multidimensional scaling (nMDS) with the “ordinate” function in the Phyloseq package (McMurdie & Holmes, 2013).

Finally, we specifically analyzed a subset of the data focusing on fishes, a well‐studied group in the region. This subset is unique as it allows direct comparisons with other available data. A dedicated section in the results addresses fish‐related findings, including discussions on alpha and beta diversity mirroring the full dataset. Additionally, we assess the accuracy of taxonomic assignments and compare detection rates with underwater towed video surveys.

## 3 RESULTS

Overall, we generated a total of 38.9 M sequences from 28 water samples from two different depths, two study sites, and four seasons. From these, approximately 10.9 M sequences were obtained using the 18S Universal metabarcode assay, approximately 12.9 M using the COI assay and 15.1 M using the fish 16S assay. Total number of reads, ASVs, and ASV that were not taxonomically assigned per marker can be found in Appendix 1. The percentage of ASVs that were taxonomically assigned with a posterior probability of 0.8 or higher to at least one taxonomic category varied among metabarcodes and taxonomic ranks (Table 3). Overall, taxonomic assignment was higher for the 16S rRNA fish metabarcode with 53.6% of ASVs assigned to the species level and 85%–91% of ASVs assigned to higher taxonomic ranks. For the 18S RNA metabarcode, 34.4% ASVs were assigned to the species level and 46%–87.4% ASVs were assigned to higher taxonomic ranks. COI achieved the lowest taxonomic assignment rate with 23.9% of ASVs assigned to the species level and 25%–32% of ASVs assigned to higher taxonomic ranks.

**TABLE 3:** Number of amplicon sequence variants (ASVs) reconstructed per metabarcode and the percentage of taxonomic assignments with a BCLA confidence score of 0.8 or higher to different taxonomic ranks.

	Number of ASVs	Phylum (%)	Class (%)	Order (%)	Family (%)	Genus (%)	Species (%)
COI	4338	32.2	30.0	30.0	27.6	25.6	23.9
18S rRNA	1859	49.1	87.4	62.6	55.4	46.4	34.4
16S rRNA	459	91.0	91.0	82.3	85.6	85.2	53.6

**FIGURE 2:**
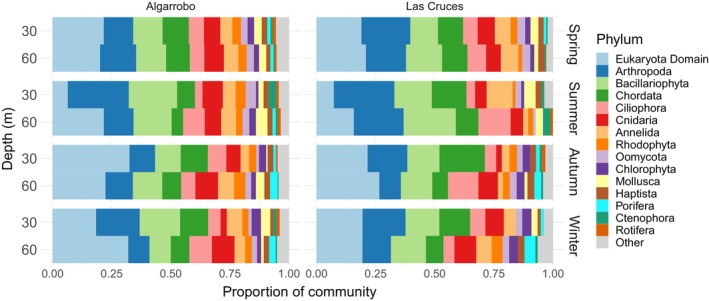
Relative family richness for the 15 most abundant phyla by season, depth (30 and 60 m), and site (Algarrobo, Las Cruces). Phyla are ordered from those with the most (left) to the least number of families (right). The “Eukaryota Domain” category corresponds to taxa that do not have a phylum assignment in NCBI taxonomy. A figure that details the richness within classes of this category can be found in Appendix 2. The remaining phyla were all pooled and are shown as “other.” *N* = 2 except for winter, where *N* = 1.

After all filtering and decontamination steps, the 18S metabarcoding data assigned ASVs to 34 phyla, 71 classes, 144 orders, and 186 families. The COI assay, in turn, assigned ASVs to 32 phyla, 63 classes, 163 orders, and 281 families. The 16S fish assay assigned ASVs to 17 families of teleost fishes, 18 genera, and 17 species. All assays combined, our data set assigned 42 phyla, 90 classes, 237 orders, and 402 families. Generally, the phyla with the highest family richness were Arthropoda, Bacillariophyta, Chordata, and Ciliophora. Figure 2 displays the relative richness of families within the 15 most diverse phyla, categorized by season, depth, and sampling site.

### 3.1 Alpha diversity results

**FIGURE 3:**
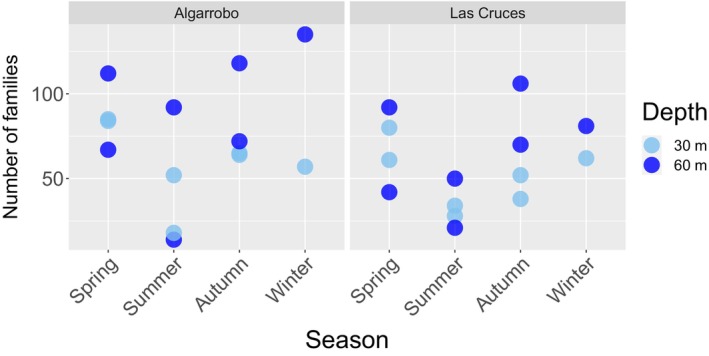
Distribution of family richness per site, season, and depth for all meta barcodes combined.

Overall, our results indicate that family richness (all metabarcoding assays combined) did not differ significantly between sites (Kruskal–Wallis chi‐squared = 1.539, df = 1, *p*‐value = .214), or depth (Kruskal–Wallis chi‐squared = 3.379, df = 1, *p*‐value = .066). Family richness was significantly lower in the summer compared to the other seasons (Figure 3) (Kruskal–Wallis chi‐squared = 9.542, df = 3, *p*‐value = .023, only the pairwise comparison between summer and spring was significant, adjusted *p* = .03, Dunn test). Variability among sampling times within each season was generally equal to or greater than variability across locations. A similar trend was observed when family richness was evaluated separately for each of the eight most abundant phyla. We found that family richness varied among seasons in five of eight phyla, significant differences in family richness between depths were only significant for the phyla Bacillariophyta and Ciliophora, and no statistical differences were observed between sites for any of the eight phyla analyzed (Appendix 3). Differences among seasons were characterized by a reduction in family richness in summer compared to other seasons (Appendix 4a) and an increase in richness with depth for Bacillariophyta and Ciliophora (Appendix 4b).

### 3.2 Community structure

**FIGURE 4:**
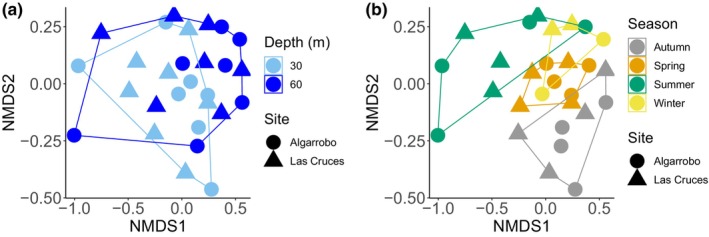
Non‐metric multidimensional scaling plot for the complete data set (combination of the three metabarcoding assays: COI, 18S, and 16S) using a Bray–Curtis dissimilarity matrix (dimensions = 2, stress = 0.145). (a) Different depths and sites. (b) Seasons and sites.

Based on the combination of the three metabarcoding assays and PERMANOVA tests, we found that season influenced the compositional structure of communities (Pseudo‐*F* = 3.311, df = 3, *p* = .001). In contrast, there were no significant differences in composition between sampling sites (Pseudo‐*F* = 1.142, df = 1, *p* = .282) or depth (Pseudo‐*F* = 1.758, df = 1, *p* = .069) (Figure 4). The statistical difference for the season was driven by the summer and autumn samples that appeared to be different from the rest. To further test if specific taxonomic groups drove these patterns, we repeated the PERMANOVA and nMDS ordinations for each of the eight most diverse phyla separately. In general, we found that the season influenced the compositional structure of communities for all eight phyla, depth had a significant influence only for Ciliophora, and the effect of site was non‐significant for all phyla (Appendix 5). nMDS ordination representations indicated that for most phyla the statistical effect of season is consistent with the general trend (Appendix 6).

### 3.3 Fish assemblage diversity

**FIGURE 5:**
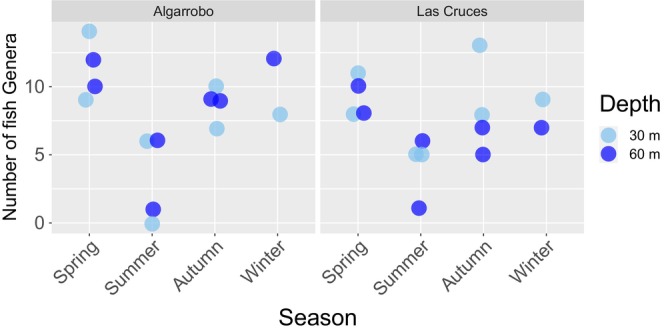
Distribution of fish genera richness among different sampling sites, seasons, and depths.

A total of 412 ASVs assigned to the Actinopterygii class were recovered with the 16S rRNA assay, 11 with the COI assay and 8 with the 18 s assay. Together these ASVs were assigned to 11 orders, 17 families, 19 genera, and 22 species. Details of taxonomic assignments to genus level per marker can be found in Appendix 7. At the genus level, 17 of the 19 assigned genera are known to be present in the coastal waters of Central Chile. One genus, *Sprattus*, is exclusively found in the southern coastal waters of Chile (south of 42° S), while one is not present in Chilean waters (*Heteroclinus*). The number of fish genera detected from eDNA water samples varied from zero to 19 per sample. The average number of fish genera was not significantly different between depths (Kruskal–Wallis chi‐squared = 0.259, df = 1, *p*‐value = .610) or sites (Kruskal–Wallis chi‐squared = 0.214, df = 1, *p*‐value = .643), but differed significantly between seasons (Kruskal–Wallis chi‐squared = 16.802, df = 3, *p*‐value <.001) (Figure 5). Pairwise comparisons between different seasons indicated that genera richness is significantly lower in summer compared to all other seasons (*p*‐values <0.05, Kruskal–Wallis multiple comparison test).

**FIGURE 6:**
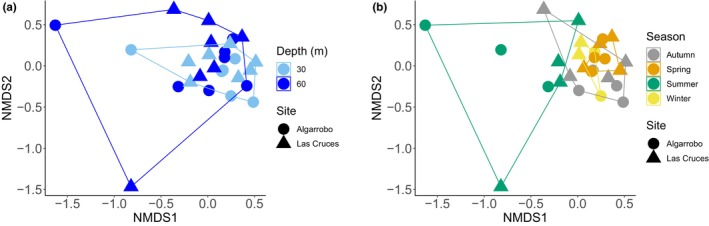
Non‐metric multidimensional scaling plot for fishes (16S metabarcoding assay) using a Bray–Curtis dissimilarity matrix (dimensions = 2, stress = 0.148). (a) Different depths and sites. (b) Seasons and sites.

According to the nMDS analyses and PERMANOVA tests, we found that the fish assemblage did not differ between sites (Pseudo‐*F* = 0.937, df = 1, *p* = .492), or depths (Pseudo‐*F* = 0.587, df = 1, *p* = .811), but differed significantly among seasons (Pseudo‐*F* = 4.032, df = 3, *p* = .001), (Figure 6).

**FIGURE 7:**
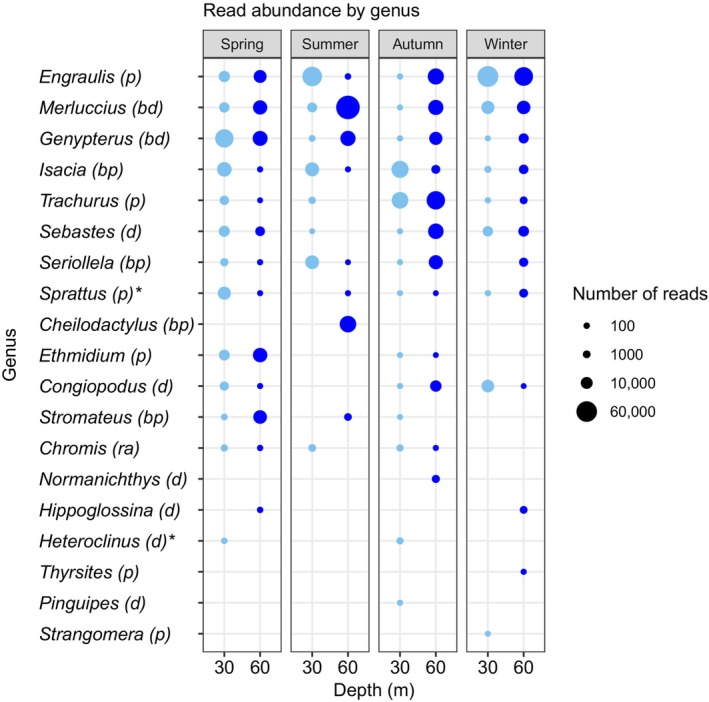
Read the abundance of eDNA sequences assigned to fish genera. Fish genera are ordered from the overall most abundant genus (top) to the least abundant one (bottom). The asterisk (*) indicates eDNA assignments that are most likely wrong at the genus level. See text for details. Letters in parentheses: pelagic (p), bathydemersal (bd), demersal (d), benthopelagic (bp), and reef‐associated (ra).

We also looked at the distribution of the abundance of reads assigned to different fish genera as a function of depth and season averaged across sampling times per season (Figure 7). Only six of the 19 assigned genera were pelagic fishes. Among the top 10 most abundant fish genera six are common benthic/demersal or benthopelagic temperate reef fishes: Merluccius (Chilean hake), *Genypterus* (Conger), *Isacia*, *Sebastes*, *Seriollella*, and *Cheilodactylus* (according to Eschmeyer's 2023 catalog of fishes [http://researcharchive.calacademy.org/research/ichthyology/catalog/fishcatmain.asp], its current name is *Chirodactylus* but we have left its previous name as it is the one that is currently in NCBI) (Figure 7). Seven genera were only present in either deep (60 m) or shallow (30 m) samples. *Cheilodactylus*, *Normanichthys*, *Hippoglossina*, and *Thyrsites* were only found in samples from 60 m depth, while *Heteroclinus*, *Pinguipes*, and *Strangomera* were only found in samples from 30 m depth.

## 4 DISCUSSION

The present study provides a first attempt to comprehensively describe the eukaryotic biodiversity of temperate mesophotic reefs and their temporal variability in central Chile, using eDNA. To our knowledge, this is the first study in mesophotic reefs in any of the large upwelling ecosystems of the world (Chavez & Messié, 2009), which, despite its limitations, makes it a baseline for future studies. Here, we show that the alpha diversity and the composition of marine eukaryotes based on eDNA taken from water samples were similar across sites separated by 16 km, and between depths (less than 2 km apart cross‐shore), but differed significantly among survey months, which were conducted during different seasons. The most different community composition, for all groups and for the better‐resolved fish assemblage, occurred in summer compared to the other seasons. Below, we discuss (a) possible drivers of observed patterns, (b) the apparent efficiency of eDNA to detect fish diversity with respect to other visual methods, and (c) the limitations and the conditions under which eDNA can become a standardized method for monitoring biodiversity in mesophotic habitats.

Our results indicate that community changes in composition could not be detected between sites that are 16 km apart, at least at the level of resolution we were able to attain. Other studies in temperate rocky reefs have shown that eDNA can detect fine‐scale structure in fish assemblages among nearshore rocky reefs and kelp forests of the coast of California (Lamy et al., 2021). Our results suggest that in central Chile, community composition is comparatively homogeneous alongshore, at least at the scale of tens of kilometers. However, we acknowledge that our sampling design is limited in this regard, and extending the geographic breadth is required to better characterize geographic alongshore variability in this region better. Further, comparing multiple sites of contrasting near‐shore oceanographic conditions (e.g., upwelling) may offer insight into the depth‐dependence of such processes in redistributing environmental conditions alongshore at depth as well as structuring mesophotic assemblages. Nevertheless, this can be logistically quite challenging considering the strong wind conditions and surge that typically prevail in these coastal areas. Alternatively, methodological limitations and uncertainties of the eDNA approach may compromise our ability to resolve spatial patterns at relatively small scales due to the dynamics of molecule decay and movement in this system (Barnes & Turner, 2016). Interestingly, we found that community composition varied very little as a function of depth when all identified taxa were considered or when different phyla were considered separately. A previous study conducted in shallow temperate kelp habitats (10–30 m depth) showed that eDNA can distinguish fish assemblages from different habitats across a depth gradient of only 20 m even when in close proximity (less than 100 m) (Port et al., 2016). However, that study was conducted in Monterey Bay, California, within comparatively well‐lit depths and crossing the seasonal thermocline, which typically imposes sharp changes in marine pelagic and benthic communities and productivity. Our study compared mesophotic depths well below the seasonal thermocline (Figure 1g), and it may represent a much smoother environmental gradient than that encountered in shallow habitats. In fact, the long‐term (annual) mean temperature at 30 m is less than 0.5°C higher than the long‐term mean at 60 m deep (see Figure 1e). In the only two mesophotic eDNA studies that we know of, Muff et al. (2023) did find a decreasing significant depth gradient in fish diversity between 60 and 140 m deep in the Mediterranean Sea (temperate) and La Perouse (tropical island). Hoban et al. (2023) on the other hand, observed marked community differences in a tropical island across depths for eukaryotes and metazoans, in particular around 45–60 m, but not for fishes. To what extent differences between our study in central Chile and these others represent differences between mesophotic reef ecologies or differences in fish fauna between oceans needs to be assessed.

Community composition changed drastically among surveys conducted in different seasons. We found that samples taken in the summer differed significantly from the rest, a consistent pattern when looking at the entire assemblage and when the analysis was restricted to different phyla and fishes. In general, it seems that these differences in composition are also associated with a reduction in species diversity detected in the summer compared to other seasons. Previous studies in tropical and subtropical coastal waters have shown significant seasonal changes in composition captured with eDNA from superficial waters (Berry et al., 2019; Djurhuus et al., 2020; Zamani et al., 2022). A recent study using remote underwater videos (RUVs) from Northern Chile also found significant seasonal changes in fish diversity in shallow reefs (Gres et al., 2024). Here, we show that this temporal variation in community structure is maintained across depths down to 60 m, and that differences in composition are also accompanied by a reduction in family richness during the summer. While the seasonality in upwelling events is well described in central Chile (Letelier et al., 2009; Tapia et al., 2014; Weidberg et al., 2020), variability in eDNA signatures is only now being reported. Upwelling carries fluctuations in physical and chemical water properties, including nutrient availability, suspended matter in the water column, pH, dissolved oxygen, and surface water temperature. At mesophotic depths, temperature does not exhibit strong seasonal variation (see Figure 1). Since primary producers are light‐limited, seasonal variation in nutrient availability is unlikely to play an important role in community composition and diversity. Nutrient concentrations are highly variable over synoptic timescales (few days) in the Humboldt upwelling coastal ecosystem (Wieters et al., 2003), and while seasonal variability is observed, nutrient concentrations remain extremely high during winter in comparison with tropical or non‐upwelling systems, typically exceeding 15 μm/L (Chavez & Messié, 2009; Nielsen & Navarrete, 2004; Wieters et al., 2003). This means that despite this variation, nutrient availability is unlikely to be a limiting factor for many photosynthetic organisms (Jacob et al., 2018). Importantly, while nutrient pulses do alter phytoplankton community composition (relative abundances, e.g., Ferreira et al., 2020) and some macroalgal growth rates (Nielsen & Navarrete, 2004), no studies in Chile (or California) have unambiguously reported that seasonal variation in nutrients can propagate up the local food webs to alter species composition (through direct observations or eDNA), at least not in the better studied shallow subtidal and intertidal communities. Therefore, one of the factors that could affect the composition of invertebrates and especially fish assemblages is the frequent occurrence of hypoxic events at waters deeper than 30 and especially 60 m, that is the penetration of cold water with an oxygen level below 2 μm/L, especially in spring and summer months (De La Maza & Farías, 2023). Sensitivity to hypoxia varies widely among marine taxa (Diaz & Rosenberg, 1995; Miller et al., 2002; Rosenberg et al., 1991). Thus, one hypothesis behind observed temporal fluctuations in diversity regards dissolved oxygen. Indeed, recent measures taken at these sites have revealed long and recurrent periods of hypoxia during spring and summer, even at shallow depths in the study region (unpublished data). To our knowledge, only a few reports have assessed temporal changes of benthic communities in northern temperate mesophotic ecosystems that go through seasonal hypoxic periods, and both reported a mortality‐associated reduction in benthic diversity during summer anoxic periods (McAllen et al., 2009; Micaroni et al., 2021). While our sampling resolution is low to make any further conclusions, these results open a new avenue to test if and how recurrent hypoxia events affect community structure and diversity of temperate mesophotic reefs in coastal upwelling systems. Further studies will also be needed to understand the nature (e.g. movement or mortality) of the seasonal changes we have documented.

In general, more than 50% of the ASVs from our eDNA samples could not be assigned to the species level with high confidence, and even at the Phylum level between 8% and 38% of sequences remained unassigned. Overall, these rates are consistent with what has been reported in the literature in other studies that have used the same assays (DiBattista et al., 2020; Marques et al., 2021) and highlight the importance of continuing ongoing efforts to improve the number of species' genetic barcodes present in public catalogs. For instance, regarding eDNA sequences assigned to fishes, all wrong assignments (assignments to species that have not been reported to this region) were due to missing reference sequences in the databases. We noted that assignments to the genera *Heteroclinus*, which belongs to the family Clinidae, most likely correspond to the genus *Myxodes*, the sole genus of this family present in Chile with three species (*M. viridis*, *M. ornatus*, and *M. cristatus*). We also noted that sequences assigned to *Sprattus* most likely correspond to the genus *Strangomera*, the latter without reference sequences for 16S in public databases, but phylogenetically close to the northern hemisphere *Sprattus* (Canales‐Aguirre et al., 2021). Finally, some of the unassigned ASVs may correspond to undescribed mesophotic species. Despite these limitations, our study shows that with relatively little field effort, eDNA provides a good approach to characterize the biodiversity of mesophotic reefs.

We are just beginning to explore Chilean mesophotic reefs, therefore, it is important to note that there is little available data that can be used to compare our results. Nevertheless, a recent study (Campoy et al., 2023) explored the diversity in the Chilean central coast (at Algarrobo and Las Cruces) using towed underwater videos (TUVs), focusing mostly on fish. Thirty‐two percent of the genera identified with eDNA were also observed using TUVs, eight genera reported with TUVs were not present in our eDNA data, and 68% of the fish identified with eDNA were not observed with TUVs. Interestingly, all genera present in TUVs and absent in eDNA lack a reference sequence in our database. In terms of depth range, *Sebastes* and *Chromis* appear in deep and shallow eDNA samples and were recorded in TUVs at those depths. In contrast, *Cheilodactylus* was only recorded at shallow depths in TUVs (9.9–28.1 m) but appeared only in the 60 m deep‐water samples. This suggests that *Cheilodactylus* may have extended bathymetrical distributions beyond those reported by depth range databases or literature, as documented in other mesophotic fish assemblages through eDNA assessments (Hoban et al., 2023; Muff et al., 2023). Conversely, *Pinguipes* was observed in both shallow and deep reefs in TUVs (8.5–81.3 m) but only in 30 m shallow eDNA water samples. To our knowledge, neither *Pinguipes chilensis* nor *Cheilodactylus variegatus* (unique species for both genera in central Chile) exhibit depth segregation, at least within the evaluated range (down to 60 m). Moreover, *P. chilensis* is one of the most abundant species occurring at intermediate depths (20–40 m) in relatively high densities (Campoy et al., 2023). Therefore, these depth distribution ranges must be taken cautiously when interpreting eDNA signals for cases with only one depth/season occurrence. As for other genera with only one depth/season occurrence and few read counts (e.g., *Strangomera* and *Thyrsites*), we lack site‐specific information for comparison. However, these two are pelagic species, and we hypothesized that eDNA samples might have just detected signals of shoals in transit during sampling or hours/days before sampling, and they are not part of the resident fish assemblage. Overall, these results are consistent with other studies that indicate that video and eDNA approaches are complementary and that eDNA metabarcoding can detect more species than TUVs (Muff et al., 2023; Polanco Fernández et al., 2021).

Our results contribute to one of the current debates regarding the type of samples (sediment or water samples) that should be used to target benthic communities (Antich et al., 2020). Water samples are usually logistically easier to obtain than sediment samples. Sediment represents a major technical challenge for deep rocky reefs, especially if a non‐destructive monitoring scheme is envisioned. Based on fish diversity captured with eDNA, our results indicate that eDNA from water samples taken at 1 m from the bottom effectively captures demersal fish species known in the area. However, a direct comparison with eDNA from sediment samples is needed to test this further. In addition, we acknowledge that the interpretation of bathymetric distributions based on eDNA must be taken cautiously because the life of eDNA in seawater and variable conditions is unknown. Indeed, eDNA might be deposited into deeper water via passive settling (e.g., as part of marine snow), through turbulent eddies and downwelling processes, and it can be advected alongshore by currents at distances yet undetermined (Barnes & Turner, 2016), potentially from shallow reefs. Therefore, more studies need to be conducted and environmental conditions reported to build a better understanding of the seascape of eDNA. The information must also be crosschecked by other sampling techniques in the future, including direct observations through technical diving or baited remote underwater videos (BRUVs), among others. Despite these limitations, our results add to the growing evidence that metabarcoding eDNA is advantageous for monitoring biodiversity in fragile, hard‐to‐visit marine habitats, including mesophotic reefs. The non‐destructive nature, repeatability, and safety of survey procedures and the decreasing costs of DNA analyses can make eDNA the method of choice for agencies interested in long‐term biodiversity monitoring. Our study thus provides a baseline for mesophotic reef biodiversity in the region.

## AUTHOR CONTRIBUTIONS

**Pablo Saenz‐Agudelo:** Conceptualization (lead); data curation (lead); formal analysis (lead); funding acquisition (equal); investigation (equal); methodology (equal); project administration (equal); writing – original draft (lead); writing – review and editing (lead). **Paula Ramirez:** Data curation (lead); formal analysis (equal); investigation (equal); methodology (equal); writing – original draft (supporting); writing – review and editing (supporting). **Ricardo Beldade:** Conceptualization (equal); formal analysis (equal); funding acquisition (equal); investigation (equal); project administration (equal); writing – original draft (supporting); writing – review and editing (equal). **Ana N. Campoy:** Formal analysis (supporting); investigation (supporting); methodology (supporting); writing – review and editing (supporting). **Vladimir Garmendia:** Formal analysis (supporting); investigation (supporting); methodology (supporting); writing – review and editing (supporting). **Francesca V. Search:** Methodology (supporting); visualization (supporting); writing – review and editing (supporting). **Miriam Fernández:** Conceptualization (equal); funding acquisition (equal); investigation (equal); project administration (equal); writing – original draft (supporting); writing – review and editing (equal). **Evie A. Wieters:** Conceptualization (equal); funding acquisition (equal); investigation (equal); project administration (equal); writing – original draft (supporting); writing – review and editing (equal). **Sergio A. Navarrete:** Conceptualization (equal); funding acquisition (equal); investigation (equal); project administration (equal); writing – original draft (supporting); writing – review and editing (equal). **Mauricio F. Landaeta:** Conceptualization (equal); formal analysis (equal); funding acquisition (equal); investigation (equal); writing – original draft (supporting); writing – review and editing (equal). **Alejandro Pérez‐Matus:** Conceptualization (equal); funding acquisition (equal); investigation (equal); methodology (equal); project administration (lead); resources (equal); supervision (equal); writing – original draft (supporting); writing – review and editing (equal).

## CONFLICT OF INTEREST STATEMENT

The authors declare that they have no conflict of interest.

## Data Availability

All demultiplexed sequence data and raw ASV tables used in this study have been deposited at Dryad and are publicly available in the following link: https://doi.org/10.5061/dryad.gqnk98sv8

## APPENDIX 1

### 1.1

Metabarcoding sequence information per sample (each sample corresponds to a pool of DNA from three water filters each amplified via two‐step PCR). See Table 1 for sampling location details. The number of reads refers to the total number of reads after demultiplexing. Post‐QF reads refer to the number of reads that passed quality filtering and were successfully assigned ASVs. The number of ASVs is also indicated. The number in brackets refers to the number of ASVs that failed to align to any of the sequences in the reference catalogs.

**Table:**

Code	Number of reads			Post‐QF reads			Number of ASVs		
	16S	COI	18S	16S	COI	18S	16S	COI	18S
ALG30_V1	15,186	563,968	286,764	6321	197,368	81,927	10 (0)	109 (83)	55 (4)
ALG60_V1	31,958	533,172	399,808	13,674	190,691	111,930	6 (3)	257 (374)	185 (4)
LC30_V1	633,258	499,398	271,496	266,987	176,848	58,507	18 (2)	54 (22)	51 (5)
LC60_V1	1,188,280	252,282	489,216	532,783	92,692	118,141	34 (4)	53 (53)	140 (8)
ALG30_V2	30	560,306	307,670	8	172,877	53,526	0 (1)	46 (14)	29 (1)
ALG60_V2	1,408,728	394,034	265,044	516,803	74,497	51,919	24 (0)	15 (6)	16 (0)
LC30_V2	872,442	457,624	967,466	341,891	110,808	170,352	19 (1)	92 (23)	43 (12)
LC60_V2	274	418,892	342,042	36	110,598	62,777	2 (0)	44 (26)	20 (5)
ALG30_OT1	687,430	574,474	496,470	293,148	225,572	157,384	47 (6)	101 (90)	113 (3)
ALG60_OT1	686,908	789,648	544,220	301,927	328,216	164,524	61 (2)	373 (444)	186 (9)
LC30_OT1	71,548	438,648	429,160	30,827	176,996	149,532	38 (2)	66 (48)	51 (2)
LC60_OT1	1,977,254	642,064	551,580	881,431	277,778	174,135	41 (0)	94 (156)	244 (9)
ALG30_OT2	401,034	375,330	484,554	173,652	139,171	162,951	41 (7)	97 (91)	97 (6)
ALG60_OT2	514,454	483,850	404,056	220,749	184,065	104,882	49 (4)	127 (119)	150 (8)
LC30_OT2	479,200	383,058	559,608	210,023	139,806	209,810	29 (1)	67 (54)	112 (12)
LC60_OT2	453,792	436,530	396,010	203,228	157,154	113,646	30 (3)	234 (229)	205 (15)
ALG30_INV	449,048	452,378	254,250	209,333	182,374	86,522	18 (0)	86 (57)	109 (8)
ALG60_INV	792,640	411,990	406,664	344,789	163,572	118,906	54 (6)	397 (752)	451 (21)
LC30_INV	834,778	389,920	305,138	371,634	160,152	97,044	47 (2)	75 (65)	148 (6)
LC60_INV	723,494	394,420	281,504	325,634	161,854	89,750	46 (2)	202 (225)	130 (4)
ALG30_PR1	150,286	497,206	398,500	68,408	184,579	106,622	23 (3)	208 (90)	163 (12)
ALG60_PR1	310,832	495,878	310,856	136,615	200,840	79,983	34 (2)	120 (55)	155 (5)
LC30_PR1	660,144	481,868	386,776	288,750	154,127	102,339	39 (1)	105 (33)	142 (4)
LC60_PR1	1488	488,668	398,200	554	192,053	117,852	12 (2)	172 (115)	248 (16)
ALG30_PR2	482,676	340,134	223,752	206,810	130,676	71,792	48 (6)	224 (144)	101 (3)
ALG60_PR2	537,660	390,368	279,532	232,391	154,018	93,465	44 (2)	335 (361)	128 (6)
LC30_PR2	650,052	376,754	243,786	282,303	145,330	77,229	58 (1)	163 (133)	101 (5)
LC60_PR2	132,662	345,186	253,224	62,062	137,221	77,059	23 (7)	76 (79)	94 (5)
Average	540,983	459,573	390,620	232,956	168,640	109,447			
Total	15,147,536	12,868,048	10,937,346	6,522,771	4,721,933	3,064,506			
Negative controls (*n* = 39)									
Average	685	4475	50,198	222	1511	13,949			
Total	26,708	174,510	1,957,724	7782	55,889	544,011			

## APPENDIX 2

### 2.1

Relative family richness for the 15 most abundant classes within the Eukaryota Domain is shown in Figure 2 separated by season, depth (30 and 60m), and site (Algarrobo, Las Cruces). Classes are ordered from those with the most (left) to the least number of families (right).

## APPENDIX 3

### 3.1

Results of Kruskal–Wallis sum rank test testing for differences in family richness among sites, depths, and seasons for each of the eight most abundant phyla. * The Eukaryota Domain category refers to taxa without a phylum assignment in the NCBI taxonomy. See Appendix 2 for details.

**Table:**

Phylum	Depth (1df)		Season (3df)		Site (1df)	
	Chi^2^	*p*	Chi^2^	*p*	Chi^2^	*p*
Eukaryota Domain*	2.30	.129	11.14	.011	1.17	.280
Arthropoda	0.45	.503	13.09	.004	0.12	.729
Bacillariophyta	5.80	.016	1.48	.686	1.60	.206
Chordata	0.12	.729	16.96	.001	0.45	.503
Cilliophora	5.93	.015	0.19	.980	0.41	.524
Cnidaria	3.26	.071	7.10	.069	0.47	.495
Annelida	3.77	.052	8.71	.033	0.92	.338
Rhodophyta	2.30	.129	11.14	.011	1.17	.280

## APPENDIX 4

### 4.1

Distribution of family richness per season (a) and depth (b) for the eight most abundant phyla.

## APPENDIX 5

### 5.1

Results of PERMANOVA analyses of Bray–Curtis dissimilarity matrices performed separately for each of the eight most diverse phyla in our dataset. *p*‐Values are estimated from 999 permutations. * The Eukaryota Domain category refers to taxa without a phylum assignment in NCBI taxonomy. See Appendix 2 for details.

**Table:**

Phylum	Depth (1df)		Season (3df)		Site (1df)	
	Pseudo‐*F*	*p*	Pseudo‐*F*	*p*	Pseudo‐*F*	*p*
Eukaryota Domain*	1.782	.080	3.795	.001	1.229	.242
Arthropoda	1.403	.200	2.206	.003	1.071	.395
Bacillariophyta	1.992	.056	2.874	.001	1.349	.246
Chordata	1.307	.283	4.149	.001	0.598	.671
Cilliophora	2.352	.033	3.087	.002	1.063	.406
Cnidaria	2.124	.063	3.201	.001	1.281	.255
Annelida	2.135	.075	2.912	.001	1.864	.100
Rhodophyta	0.793	.547	6.932	.001	0.706	.619

## APPENDIX 6

### 6.1

Non‐metric multidimensional scaling plot for each of the eight most diverse phyla (see Figure 2 for details) using a Bray–Curtis dissimilarity matrix and colored by season

## APPENDIX 7

### 7.1

Taxonomic assignments to genus and species level with a BCLA confidence score of 0.8 or higher for each of the three assays implemented in this study. * After the species denotes wrong species assignment but correct genus assignment. * After the genus denotes the wrong genus and species assignment. Incorrect assignments were determined based on known geographic distributions for these species obtained from Fishbase and local surveys.

**Table:**

16s rRNA assay	18s rRNA assay	COI assay	Comments
*Engraulis ringens*		*Engraulis ringens*	Visual surveys (Pérez‐Matus et al., 2007, 2022)
*Genypterus*		*Genypterus chilensis*	Visual surveys (Pérez‐Matus et al., 2007, 2022)
*Merluccius gayi*	*Merluccius merluccius**	*Merluccius gayi*	*M. gayi* is absent in the 18S reference database. Baited Remote Underwater Video (unpublished data)
*Isacia conceptionis*		*Isacia conceptionis*	Visual surveys (Pérez‐Matus et al., 2007, 2022)
*Chromis enchrysura**		*Chromis enchrysura**	Most likely *C. crusma*, but *C. crusma* is absent from all databases. Visual survey (Pérez‐Matus et al., 2007, 2022), TUV (Campoy et al., 2023)
*Trachurus symmetricus**		*Trachurus murphyi*	*T. symmetricus* absent from 16s database. Visual surveys (Pérez‐Matus et al., 2007, 2022)
*Sprattus muelleri**			Most likely *Strangomera bentiki*, absent from 16s database. FishBase
*Cheilodactylus*			FishBase
*Seriolella*		*Seriolella brama**	Likely *S. violacea*, absent from 16s database. Low resolution of the COI metabarcode. Visual surveys (Pérez‐Matus et al., 2007, 2022). TUV (Campoy et al., 2023)
*Sebastes oculatus*	*Sebastes umbrosus**		Low resolution of the 18s metabarcode. Visual surveys (Pérez‐Matus et al., 2007, 2022). TUV (Campoy et al., 2023)
*Congiopodus peruvianus*			Visual surveys (Pérez‐Matus et al., 2007, 2022)
*Normanichthys crockeri*			FishBase
*Stromateus stellatus*			FishBase
*Hippoglossina stomata**			Likely *H. macrops*, absent from 16s database. FishBase.
*Heteroclinus** *heptaeolus /wilsoni*			Most likely *Myxodes* spp., absent from 16s and COI databases. Visual surveys (Ruz et al., 2021)
*Thyrsites atun*			Visual surveys (Pérez‐Matus et al., 2007, 2022)
		*Pinguipes brasilianus**	Most likely *P. chilensis*, absent from all databases. Visual surveys (Pérez‐Matus et al., 2007, 2022), TUV (Campoy et al., 2023)
*Ethmidium maculatum*			FishBase
		*Strangomera bentincki*	FishBase
